# DNA unwinding assay using streptavidin-bound oligonucleotides

**DOI:** 10.1186/1471-2199-7-43

**Published:** 2006-11-28

**Authors:** Jae-Ho Shin, Zvi Kelman

**Affiliations:** 1 University of Maryland Biotechnology Institute, Center for Advanced Research in Biotechnology, 9600 Gudelsky Drive, Rockville, MD 20850, USA

## Abstract

**Background:**

Helicases play essential roles in many cellular processes including replication, transcription and translation. Most helicases translocate along one strand of the duplex while displacing the complementary strand (of either DNA or RNA). Thus, helicases have directionality. They move along nucleic acids in either the 3'→ 5' or 5'→ 3' direction. The directionality of helicases with low activity or of those that cannot initiate duplex unwinding from a substrate that contains only one single-stranded overhang region is difficult to determine.

**Results:**

An improved assay to determine helicase directionality was developed that uses a substrate containing biotinylated oligonucleotides. As a proof of concept, it was shown that the substrates substantially improve helicase activity and directionality determination for several DNA helicases in comparison to more traditional substrates. In addition, a universal substrate that can be used to determine the directionality of both 3'→ 5' and 5'→ 3' helicases was developed.

**Conclusion:**

It is shown here that the use of a biotin-streptavidin complex as a helicase substrate improves helicase activity and the determination of helicase directionality. The method described is simpler that the currently available techniques.

## Background

Helicases play important roles in DNA and RNA metabolic processes such as replication, recombination, repair, transcription and translation [1]. The enzyme catalyzes the unwinding of duplex nucleic acid, utilizing the energy derived from nucleoside triphosphate hydrolysis to translocate along one strand of the duplex and to displace the complementary strand. Thus, most helicases have a specific polarity. They move along the strand either in the 3'→ 5' or 5'→ 3' direction. Some helicases, however, are bipolar and can move in either direction (e.g. [2]). Thus, one of the first properties to be determined for a new helicase is its directionality.

**Figure 1:**
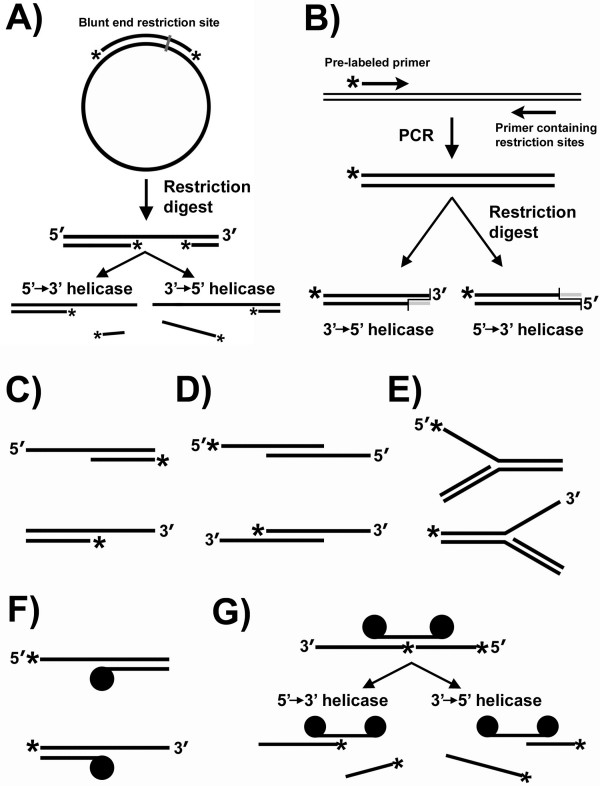
Schematic representation of different substrates used to determine helicase directionality. *, labeled strand; ●, biotin-streptavidin. See text for details.

To date, several different substrates have been used to determine helicase directionality. One such substrate is schematically shown in Figure 1A[3]. It is made by annealing a 5' ^32^P-labled complementary strand to a blunt end restriction enzyme site on a single-stranded plasmid DNA (e.g. M13 or φX174), followed by ^32^P labeling of the 3' end using ^32^P-labled dNTP and DNA polymerase. The labeled substrate is then digested with the restriction enzyme. The result is a linear molecule with a large internal region of ssDNA where a helicase can assemble, and two ^32^P-labled duplex regions of different lengths. Depending on enzyme polarity, only one of the duplex regions will be unwound in a helicase assay. The unwound product can be resolved from the starting duplex by electrophoresis followed by autoradiography. It is also possible to use this approach to make two substrates, where in each of which only one of the two short fragments is labeled [4]. Regardless if one or two substrates are used, this approach is amenable only for DNA helicases and only for those that are capable of assembly on ssDNA in the absence of an overhang region. Several helicases (e.g. *Escherichia coli *DnaB) cannot efficiently initiate unwinding on this type of substrate.

For DNA helicases that can initiate unwinding from a short (4 nucleotide) ssDNA region without a fork-like DNA structure, PCR can be used to generate a substrate for a polarity assay (Figure 1B) [5]. A PCR product is generated with ^32^P-labeled nucleotides, and then digested with restriction enzyme to produce substrates with a short 3' or 5' overhang ssDNA end. However, this approach is also only useful for DNA helicases, and only the very few that can initiate unwinding from a very short ssDNA region.

The most common substrates to determine directionality are schematically shown in Figure 1C. To produce them, a short ^32^P-labeled oligonucleotide is annealed to longer oligonucleotides, producing substrates containing either a 3' or 5' overhang region. Depending on helicase polarity only one of the substrates will be unwound. A slight modification of the substrate has also been used. In this substrate (Fig. 1D) only 3' or 5' overhang regions are present [6-8]. Although these approaches are suitable to determine the polarity of both DNA and RNA helicases they require the enzyme to be active on substrates that contain only one single-stranded overhang region. A number of helicases have extremely poor activity on these substrates (e.g. [9]) because they require a forked substrate for efficient activity. However, because a forked substrate by definition contains both a 3' and a 5' single-stranded overhang region, simple forked substrates are not suitable for directionality determination.

One approach used to bypass the requirement for a forked substrate by some helicases is shown in Figure 1E[10]. Each substrate contains a duplex region with a forked structure in which one arm is composed of ssDNA or RNA and the other arm has dsDNA or dsRNA. In each substrate the directionality of the single-stranded portion is different, either 3'→ 5' or 5'→ 3', and thus this substrate was used to determine enzyme directionality. However, as several helicases can translocate on dsDNA [11,12] the approach shown in Figure 1E cannot be used to determine the directionality of those helicases.

Here, an improvement on the technique that utilizes oligonucleotides to determine helicase directionality is described. The assay utilizes biotinylated oligonucleotides, which upon binding to streptavidin provides an efficient substrate to determine DNA and RNA helicase directionality, even those that exhibit no, or limited, activity on substrates that contain only a ssDNA overhanging region without a fork-like structure.

## Results and discussion

Helicases translocate along one strand of the duplex and displace the complementary strand. They move along the DNA or RNA in either the 3'→ 5' or 5'→ 3' direction (some can move in either direction). Knowing the directionality is important to determine the role of the helicase. For example, the directionality of the replicative helicase will dictate if it moves along the leading or the lagging strand. Therefore, determining helicase polarity is one of the characteristics determined of a newly isolated helicase. Although the determination of helicase polarity is relatively simple if the enzyme can initiate translocation from a flat substrate (one with only one single-stranded overhang region), the currently available techniques are not efficient for enzymes that require forked substrates.

It is thought that in enzymes that require forked structures, at least for the hexameric DNA helicases, the role of the non-translocating strand is to ease the exclusion of one strand from the helicase. It has been shown for several helicases that in the absence of the non-translocating overhang strand the enzyme will translocate along the duplex (for examples see: [11,12]).

A new approach to achieve strand exclusion, which depends upon biotinylated DNA or RNA oligonucleotides to determine helicase directionality, is described here. When streptavidin binds to the substrate shown in Figure 1F it functions in a manner similar to forked substrate presumably by excluding one strand from the helicase [12].

Biotinylated oligonucleotides were previously used in different nucleic acid studies. For example: streptavidin-bound oligonucleotides were used to block helicase movement along DNA [11-13], to demonstrate strand exclusion [12] or helicase translocation along duplex DNA [9,11]. However, streptavidin-bound oligonucleotides have not yet been used to determine helicase directionality.

To determine if the biotinylated substrates enhance helicase activity for enzymes that prefer forked substrates, and to determine whether they can be used to determine helicase directionality, the substrates shown in Figure 1F were tested with several DNA helicases. Three of the helicases, representing the replicative helicases from bacteria, eukarya and archaea, the *E. coli *DnaB (ecDnaB), the *Schizosaccharomyces pombe *MCM4,6,7 complex (spMCM4,6,7) and the *Thermoplasma acidophilum *MCM (taMCM), were previously shown to have weak activity on substrates lacking fork-like structures [9,14]. In addition, while the DnaB helicase possesses 5'→ 3' polarity the MCM helicases have 3'→ 5' polarity.

**Figure 2:**
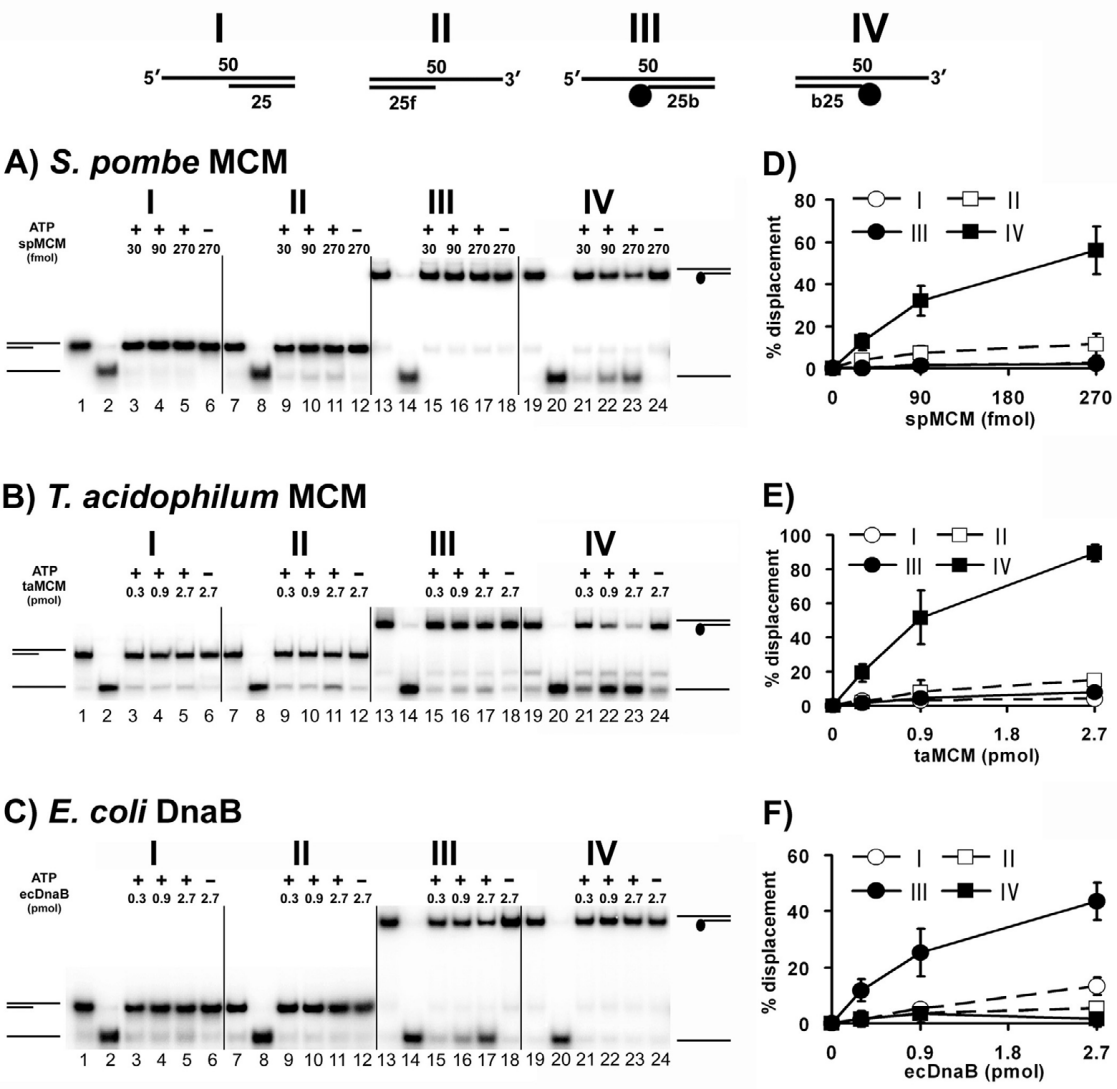
**Streptavidin-bound oligonucleotides ease the determination of helicase directionality**. Helicase directionality assays were performed as described in "Methods" using the substrates shown at the top (I-IV). In each substrate the longer oligonucleotide is ^32^P-labeled and streptavidin is marked with a closed circle. Panels A and D, spMCM; panels B and E, taMCM; panels C and F, ecDnaB. Representative results are shown (A-C) together with a summary and standard deviation of three independent experiments (D-F). The amount of protein (as hexamer, panel A, and monomer, panels B and C) used and whether ATP was present or not is indicated at the top of each panel.

As shown in Figure 2 the binding of streptavidin to the substrate substantially improves helicase activity of all three enzymes (Fig, 2A and 2B compare IV to II and C compare III to I, see also D-F). The streptavidin-bound substrates also allow for easy determination of helicase directionality. When the archaeal and eukaryal MCM proteins were used in the presence of only a 5'-overhang region no helicase activity could be detected (Fig. 2A and 2B, III). The enzymes are active only in the presence of a 3'-overhang region (Fig. 2A and 2B, IV). Similarly, the *E. coli *DnaB helicase is active only when provided with a 5'-overhang region (Fig. 2C, III) but not in the presence of only a 3'-overhang (Fig. 2C, IV). These results demonstrate that the presence of streptavidin on the substrate does not alter the directionality of the enzymes.

**Figure 3:**
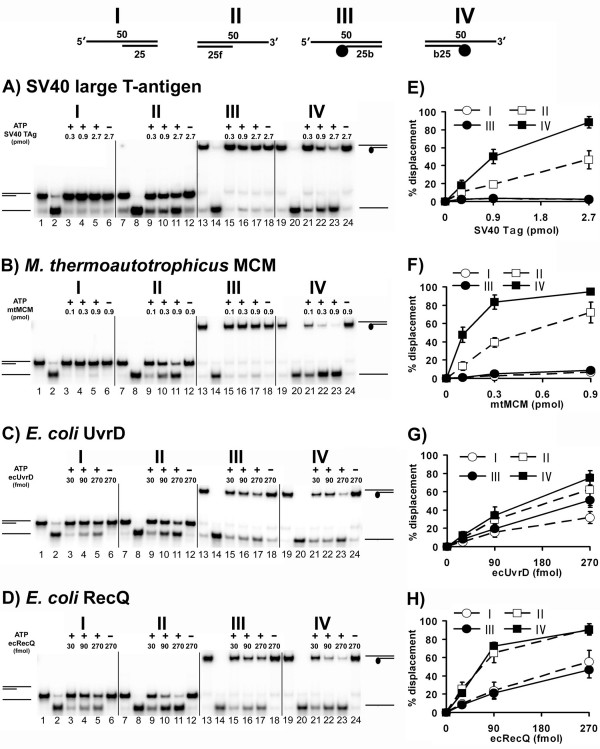
**Streptavidin-bound oligonucleotides can be used to determine the directionality of various helicases**. Helicase directionality assays were performed as described in "Methods" using the substrates shown at the top (I-IV). In each substrate the longer oligonucleotide is ^32^P-labeled and streptavidin is marked by a closed circle. Panels A and E, Tag; panels B and F, mtMCM; panels C and G, ecUvrD; panels D and H, ecRecQ. Representative results are shown (A-D) together with a summary with standard deviation of three independent experiments (E-H). The amount of protein (as monomer) used and whether ATP was present or not is indicated in the top of each panel.

Two additional replicative helicases, the viral Simian Virus 40 large T-antigen (Tag) and the MCM helicase from the archaeon *Methanothermobacter thermautotrophicus *(mtMCM), previously shown to be active on a substrate containing only a 3'-overhang region, were also evaluated with the streptavidin-containing substrates. As has been previously reported, both Tag (Fig. 3A II) and mtMCM (Fig. 3B II) efficiently unwind the substrates containing only a 3'-overhang region. However, even the activity of these helicases is enhanced in the presence of the streptavidin-bound template (Fig. 3A and 3B compare IV to II, panels E and F compare open to closed squares).

The substrates were also tested with two other *E. coli *helicases, RecQ and UvrD. As shown in Figure 3C and 3D both enzymes are active on substrates containing a 3'-overhang region whether it contains streptavidin (Fig. 3C and 3D II) or not (Fig. 3C and 3D IV). However, both enzymes also have activity, albeit limited, when the enzyme is provided with only a 5'-overhang region (Fig. 3C and 3D I and III). This is likely due to limited initiation of DNA unwinding from the blunt end side of the duplex as has previously been reported for both enzymes [15,16].

**Figure 4:**
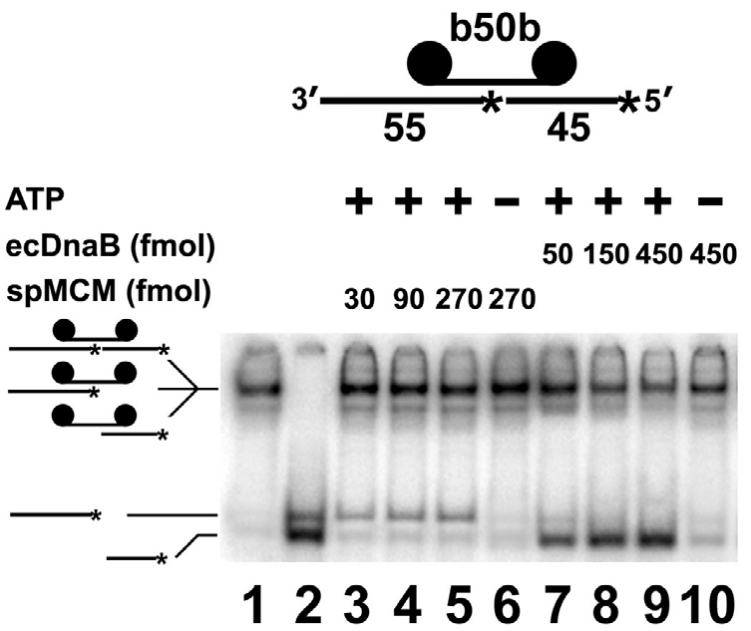
**Universal substrate to determine helicase directionality using streptavidin-bound oligonucleotides**. Helicase directionality assays were performed as described in "Methods" using the substrates shown at the top. The ^32^P-labeled oligonucleotides are marked with asterisks and streptavidin is marked with large closed circles. Representative results are shown together with a summary and standard deviation of three independent experiments. The amount of protein (as hexamer) used and whether ATP was present or not is indicated at the top of each panel.

For the experiments described in Figures 2 and 3 two substrates need to be used; one with a 3' overhang region and one with a 5' overhang region. Although this is the cleanest way to determine helicase directionality, one might prefer to use a single, universal, substrate. Therefore, the advantage of biotinylated oligonucleotide was adopted to create a universal substrate for helicase activity. The substrate, schematically shown in Figure 1G, uses two ^32^P-labeled oligonucleotides of different length hybridized to a third oligonucleotide which contains biotin at both ends. Using ecDnaB and spMCM as representative examples of 5' and 3' helicases, respectively, it was found that this substrate can be used to determine helicase directionality (Fig. 4).

Taken together, the data presented here demonstrate that the use of streptavidin-containing substrates can provide a general method to determine helicase directionality even if the helicase studied has limited activity on traditional substrates. The data also show that although the presence of streptavidin on the substrate does not alter the biochemical properties of the helicases it improves helicase activity of some and thus enables the determination of the directionality of weak helicases.

It should be noted, however, that several helicases have been shown to displace streptavidin from biotinylated oligonucleotide including the bacteriophage T4 Dda helicase [17,18], the *M. thermautotrophicus *helicase [9,11], the NS3 helicase of Hepatitis C Virus [19] and Tag [19]. These enzymes are potent and thus their directionality can be detected by more conventional methods. In addition, as shown in Figure 3B, although *M. thermautotrophicus *can displace streptavidin from biotinylated oligonucleotide its helicase activity is stimulated in the presence of streptavidin on the DNA. However, it is possible that other, not yet characterized helicases will be able to displace streptavidin from the substrates described here (Figs. 1F and 1G). For such helicases, these substrates might not be useful to determine directionality.

## Conclusion

Here an improved method to determine helicase directionality is described, which utilizes biotinylated oligonucleotide. Utilizing the approach to determine the directionality of several DNA helicases as a proof of concept it was shown that the approach is particularly useful to determine the directionality, and detect helicase activity, of helicases for which the currently available methods are not easily employed.

## Methods

### Substrate preparation for helicase assay

Oligonucleotides used for the preparation of the helicase substrates are listed in Table 1. Helicase substrates were made by labeling the oligonucleotides at the 5' end using [γ-^32^P]ATP and T4 polynucleotide kinase. The labeled oligonucleotide(s) was hybridized to the complimentary strand followed by the removal of the unincorporated [γ-^32^P]ATP and/or unannealed oligonucleotide. The detailed protocol for substrate preparation is given below.

Oligonucleotide labeling was performed as followed: 10 pmol oligonucleotide was incubated with 5 μl [γ-^32^P]ATP (~16.5 pmol, 3000 Ci/mmol, GE Biosciences), 1 μl (10 U) of polynucleotide kinase, 2 μl of 10× polynucleotide kinase buffer and H_2_O to final volume of 20 μl for 30 min at 37°C. Labeling reaction was stopped by adding 1 μl of 0.5 M EDTA to a final concentration of 25 mM. Specific activity was determined by spotting 1 μl of the labeling reaction a DE81 filter disk (Whatman). The filter was washed three times in buffer containing 0.3 M ammonium formate and 0.01 M sodium pyrophosphate to remove the unincorporated ATP. The filter was rinsed with H_2_O, then ethanol, and air dried. The radioactivity retained on the filter was determined using scintillation counting. As 1 μl of the labeling reaction mixture contain 500 fmol of oligonucleotides one can calculate the specific activity as cpm/fmol.

Oligonucleotide hybridization was performed as followed: for the substrate containing 2 oligonucleotides (Fig. 1F), 20 pmol of the biotinylated oligonucleotide was added to 10 pmol of the ^32^P-labeled oligonucleotide, resulting in a 1:2 ratio between the labeled and unlabeled oligonucleotides, together with 1 μl of 0.5 M Hepes-NaOH (pH = 7.5) and 2.5 μl of 0.5 M NaCl. For the substrate containing 3 oligonucleotides (Fig. 1G), 5 pmol of the biotinylated oligonucleotide was added to 10 pmol each of the ^32^P-labeled oligonucleotides, resulting in a 2:1 ratio between the labeled and unlabeled oligonucleotides, together with 2 μl of 0.5 M Hepes-NaOH (pH = 7.5) and 5 μl of 0.5 M NaCl. The mixture was heated at 99°C for 3 min followed by slow cooling to 22°C. The mixture was incubated at 22°C for 1 hr. After hybridization, 5 μl of 6× loading buffer containing 50% glycerol, 0.1% xylene cyanol and 0.1% bromophenol blue was added and the mixture was fractionated through an 8% native polyacrylamide gel for 1 h at 100 V in 0.5× TBE (45 mM Tris, 4.5 mM boric acid, 0.5 mM EDTA). The substrate was located by autoradiography, excised from the gel, sliced into small pieces and eluted in 3 volumes of an elution buffer [0.5 M ammonium acetate, 10 mM magnesium acetate, 1 mM EDTA (pH = 8.0)] by incubating at 37°C for 2 hr. After centrifugation, the supernatant was collected, and the gel slices were extracted once more with elution buffer. Following centrifugation, both supernatant fractions were combined, and the DNA was ethanol precipitated and dissolved in TE [10 mM Tris-HCl (pH = 7.5), 1 mM EDTA]. The specific activity of substrate was determined by liquid scintillation counting.

For assays involving biotin-streptavidin complexes, 10 fmol of biotinylated substrate was incubated with 1 pmol of streptavidin for 10 min at 30°C prior to use.

### Helicase assay

*S. pombe *MCM helicase assays were performed in 15 μl reaction mixtures containing 25 mM Hepes-NaOH (pH = 7.5), 25 mM potassium acetate, 10 mM magnesium acetate, 5 mM ATP, 1 mM DTT, 100 μg/ml BSA, 10 fmol substrate, and spMCM4,6,7 complex as indicated in Figures 2 and 4. Reactions were carried out at 30°C for 1 hr.

*T. acidophilum *and *M. thermautotrophicus *MCM helicases assays were performed in 15 μl reaction mixtures containing 20 mM Tris-HCl (pH = 8.5), 10 mM MgCl_2_, 2 mM dithiothreitol, 100 μg/ml BSA, 5 mM ATP, 10 fmol of substrate and proteins as indicated in Figure 2. Reactions were carried out at 60°C for 1 hr.

*E. coli *DnaB, UvrD, RecQ and Tag helicase assays were performed in 15 μl reaction mixtures containing 20 mM Tris-HCl (pH = 7.5), 5 mM MgCl_2_, 1 mM DTT, 100 μg/ml BSA, 2.5 mM ATP, 10 fmol substrate and proteins as indicated in Figures 2, 3, 4. Reactions were carried out at 37°C for 1 hr.

After incubation reactions were stopped by adding 5 μl of 5× loading buffer (100 mM EDTA, 0.1% xylene cyanol, 0.1% bromophenol blue, and 50% glycerol), and aliquots were fractionated on an 8% native polyacrylamide gel in 0.5× TBE and electrophoresed for 1.5 hr at 150 V at 4°C. Helicase activity was visualized and quantitated by phosphorimaging.

## Authors' contributions

JHS and ZK conceived and designed the study. JHS performed the experiments. ZK drafted the manuscript with help from JHS. All authors read and approved the final manuscript.

## Figures and Tables

**Table 1:** DNA oligonucleotides used in the study.

**Oligos**	**Sequence**
50	5'-GGGACGCGTCGGCCTGGCACGTCGGCCGCTGCGGCCAGGCACCCGATGGC-3'
25	5'-GCCATCGGGTGCCTGGCCGCAGCGG-3'
25f	5'-CCGACGTGCCAGGCCGACGCGTCCC-3'
25b	5'-GCCATCGGGTGCCTGGCCGCAGCGG-Biotin-3'
b25	5'-Biotin-CCGACGTGCCAGGCCGACGCGTCCC-3'
b50b	5'-Biotin-GGGACGCGTCGGCCTGGCACGTCGGCCGCTGCGGCCAGGCACCCG ATGGC-Biotin-3'
45	5'-TTTGTTTGTTTGTTTGTTTGGCCATCGGGTGCCTGGCCGCAGCGG-3'
55	5'-CCGACGTGCCAGGCCGACGCGTCCCTTTGTTTGTTTGTTTGTTTGTTTGTTTGTT-3'
